# Optically pumped magnetometers enhance neuroimaging performance—An EEG, OPM, and SQUID-MEG study

**DOI:** 10.1016/j.isci.2026.115489

**Published:** 2026-03-26

**Authors:** Marion Brickwedde, Paul Anders, Peter Krüger, Tilmann Sander, Peter J. Uhlhaas

**Affiliations:** 1 Physikalisch-Technische Bundesanstalt, Department for Biosignals, 10587 Berlin, Germany; 2 Charité-Universitätsmedizin Berlin, Corporate Member of Freie Universität Berlin, Humboldt-Universität Berlin and Berlin Institute of Health, Department of Child and Adolescent Psychiatry, 13353 Berlin, Germany

**Keywords:** neuroscience, sensory neuroscience, techniques in neuroscience

## Abstract

Much of our understanding of how neural circuit activity relates to human behavior and cognition stems from non-invasive assessments of the central nervous system. Further progress on key questions, however, requires measurements with a higher signal-to-noise ratio (SNR). Here, we show that in an established 40 Hz auditory steady-state paradigm, optically pumped magnetometers (OPMs) significantly outperform not only electroencephalography (EEG) but also conventional magnetoencephalography (MEG) in SNR and inter-trial phase coherence (ITPC). SNRs of OPMs were increased by up to 205% compared to EEG and by up to 40% compared to conventional MEG. As few as 10–16 OPM sensors were sufficient to significantly outperform 56 EEG electrodes. To maximize the representation of evoked responses, we employed spatial filters, which greatly enhanced SNRs across measurement modalities. Our data provide evidence for the potential of OPMs to enhance non-invasive neuroimaging, paving the way for important advances in basic as well as translational neuroscience.

## Highlights

• OPM is a recent technology that can assess human brain activity non-invasively
• We performed parallel EEG/OPM and separate SQUID-MEG recordings
• The signal-to-noise ratio of OPMs significantly outperformed EEG and SQUID-MEG
• We show that OPMs have the potential to advance non-invasive neuroimaging

## Graphical abstract

**Figure:**
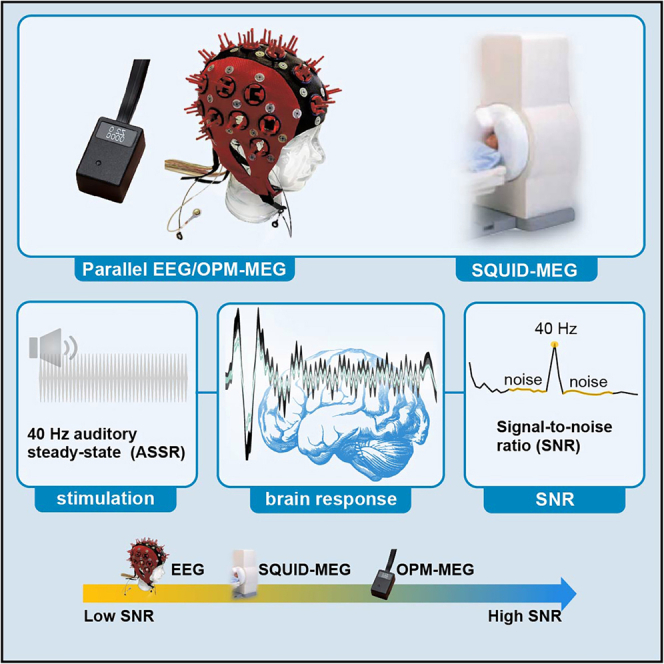

## Introduction

The measurement of neural signals with current non-invasive instruments is constrained by limited signal-to-noise ratio (SNR) and availability.^1^ Recently, optically pumped magnetometers (OPMs) were proposed as quantum sensors for magnetoencephalography (MEG), which could potentially improve SNRs compared to the current gold-standard electroencephalography (EEG) and MEG systems.^2^^,^^3^^,^^4^ Yet despite their potential, a systematic comparison between OPM, EEG, and conventional MEG that investigates SNRs across modalities is currently missing. Here, we show that SNR and trial coherence of OPMs significantly exceed EEG and conventional MEG, highlighting the potential of OPMs for non-invasive neuroimaging.

Conventional MEG systems rely on superconducting quantum interference devices (SQUIDs), which require continuous helium cooling. As a result, SQUID sensors can only be accommodated in rigid dewars that place the sensors several centimeters away from the human scalp. In contrast, OPMs measure neural signals via light absorption in vaporized alkali atoms, where the absorption is modulated by surrounding magnetic fields.^5^^,^^6^^,^^7^ Current OPM sensors are compact (volume: ∼5 cm^3^)^8^ and can therefore be positioned directly on the scalp. Because magnetic field strength decays with the square of the distance to neural sources,^9^ OPMs record neuronal signals with higher signal amplitude^3^ and detect magnetic fields that may be inaccessible to SQUID sensors. This is supported by simulations demonstrating increased signal amplitude, SNR, as well as improved dipole localization accuracy for OPMs compared to SQUID sensors.^4^^,^^10^^,^^11^^,^^12^^,^^13^

EEG signals, on the other hand, propagate through tissue with varying electrical conductivity—such as the scalp, skull, and cerebrospinal fluid—which leads to spatial smearing and mixing of neural sources at the sensor level.^14^^,^^15^

Several studies showed that OPMs can identify resting-state connectivity, event-related fields, and steady-state responses comparable to SQUIDs.^11^^,^^16^^,^^17^^,^^18^^,^^19^^,^^20^^,^^21^^,^^22^^,^^23^^,^^24^^,^^25^ In direct comparisons, there have been indications that the performance of OPMs is superior to EEG and similar to SQUID systems, but contrary results were reported as well.^11^^,^^26^^,^^27^^,^^28^ Additionally, comparisons between SQUID, EEG, and OPM systems were based on relatively small sample sizes (*n* < 10).^3^^,^^22^^,^^29^^,^^30^^,^^31^^,^^32^^,^^33^ As a result, further studies are needed for the robust assessment of the performance of OPM system relative to EEG, and SQUID systems.

To address this question, we recorded parallel EEG/OPM data and obtained SQUID measurements in a separate session from the same participants (*n* = 23, for participant details see Table 1) during 40 Hz auditory steady-state stimulation (ASSR), an established paradigm in basic as well as clinical research.^16^^,^^34^^,^^35^^,^^36^^,^^37^ To exploit the full potential of each measurement modality,^38^^,^^39^ we applied a spatial filter designed to maximize the relevant signal across sensors and electrodes.^38^^,^^39^ Our results demonstrate that OPMs significantly exceed EEG and SQUID in SNRs and phase coherence across trials.

**Table 1:** Demographic details of study participants

Age	Sex	German citizenship	Migration (generation)
34	male	yes	no
23	female	yes	no
27	male	yes	no
25	female	yes	yes (1)
28	male	yes	no
49	female	yes	no
38	male	yes	no
36	female	yes	no
45	female	yes	no
26	female	yes	no
23	female	yes	no
36	female	yes	yes (2)
49	male	yes	no
25	male	yes	no
26	female	yes	yes (2)
48	male	yes	no
39	male	yes	yes (1)
50	male	yes	no
28	female	yes	yes (2)
50	male	yes	no
41	female	yes	no
24	male	yes	no
39	male	yes	no

Note: generation of migration is denoted as individual migration (1) or parental migration (2).

## Results

### Measuring 40 Hz ASSRs with OPM, EEG, and SQUID

**Figure 1:**
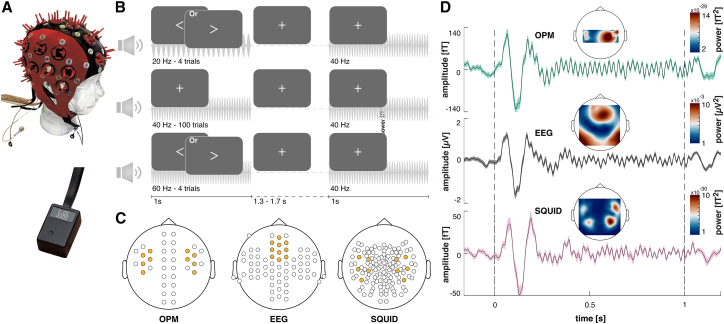
Measuring auditory steady-state potentials with EEG, OPM, and SQUID (A) EEG and OPM were measured in parallel with a custom cap (based on a commercial product by ANT-Neuro (https://www.ant-neuro.com/), which included OPMs (QuSpin, 2^nd^ Gen; https://quspin.com/) inserted into self-made holders in between EEG electrodes. (B) We presented amplitude-modulated 40 Hz tones with a duration of 1 s and a jittered inter-trial interval of 1.3–1.7 s. Participants were asked to fixate to the center of the screen and react to rare stimuli consisting of arrows pointing to the right, while ignoring arrows to the left. In trials with arrows, the tone was amplitude modulated at 60 or 20 Hz. (C) We compared the performance of 10 OPM sensors with 10 EEG electrodes and 10 SQUID gradiometers. Their position (marked in yellow) was chosen to reflect topographic equivalence of the position of SQUID and OPM sensors and, in the case of EEG, the strongest activation of 40 Hz activity. (D) OPM, EEG, and SQUID showed strong correspondence of the measured auditory steady-state response averaged over all participants (*n* = 23). Displayed is the sensor/electrode with the highest SNR for each measurement modality. Shaded areas represent mean ± SEM. Due to a reduced distance to the scalp, the signal amplitude was increased by a factor of 2–3 for OPMs compared to SQUID. The topography of the auditory steady-state response in SQUID reflects the dipolar temporal structure expected for auditory responses. The topography for OPMs suggests a similar pattern; however, the topography was constrained by the limited number of sensors. The auditory steady-state response of the EEG displayed most strongly over fronto-central electrodes.

We designed a wearable cap to allow simultaneous measurement of EEG and OPM (Figure 1A). To cover right and left temporal areas, we positioned 5 OPM sensors between EEG electrodes on each lateral side. For some participants, we had up to 6 additional Sensors available (see Table 2 for details). By choosing 10 SQUID gradiometers in matching positions to the 10 shared OPM sensors and 10 EEG electrodes that best reflect the maximum activity of ASSRs, we ensured comparability between measurement modalities (Figure 1C).

**Table 2:** Distribution of available OPM sensors during recordings

	No. of OPM sensors				
	10	11	12	14	16
No. of recordings (equivalent to no. of participants)	6	5	1	2	9

We applied 40 Hz ASSR stimulation for which the spectral and topographic responses are well established (Figure 1B).^16^^,^^34^^,^^35^^,^^36^^,^^37^ Participants received instructions to focus on the center of the screen while 40 Hz amplitude-modulated 1s tones were presented. In order to keep participants engaged, we included a rare go-nogo task, which consisted of either 20 or 60 Hz amplitude-modulated tones and an arrow which participants needed to respond to (pointing to the right) or ignore (pointing to the left).

Across measurement modalities, we found a strong similarity in the temporal course of auditory evoked potentials and steady-state responses. The signal amplitude was increased by a factor of 2–3 for OPM compared to SQUID signals (Figure 1D). The topographic distribution of auditory steady-state responses for EEG was strongest over fronto-central electrodes and over temporal areas for the SQUID measurement, which reflected previous findings.^35^^,^^36^^,^^40^

As SQUID recordings took place in a separate session and room, the shielding factor was reduced compared to the EEG/OPM recording, which may impact SNR values. However, in contrast to OPMs, SQUID systems are stationary, which reduces artifacts created by movement through magnetic background fields and therefore results in lower shielding requirements.^41^

### The SNR of OPMs exceeds EEG and SQUID

We investigated the SNR as peak power in the trial average at 40 Hz in relation to power at adjacent frequencies between 250 and 1,000 ms. To optimize the ASSR signal representation, we applied canonical correlation as a spatial filter, which maximizes the covariance between single trials and the average trial.^39^^,^^40^. To ensure comparability between measurement modalities, we kept preprocessing steps identical. While we performed artefact rejection separately for each modality, the number rejected trials and ICA components were comparable (see Table 3). We segregated the data into bins of 10 trials, simulating experimental conditions with varying trial numbers. In a two-factorial repeated measures ANOVA (3 × 9: measurement modality × trial count), measurement modalities differed significantly in SNR (*F*_(1.89,37.88)_ = 13.13; *p* < 0.001; η^2^_*p*_ = 0.40), and SNRs increased with trial numbers (*F*_(1.36,27.24)_ = 47.04; *p* < 0.001; η^2^_*p*_ = 0.70).

**Table 3:** Average removed trials, channels, and ICA components per modality

	EEG	OPM	SQUID
Trials	18 (±17)	20 (±21)	9 (±11)
ICA components	3 (±1)	3 (±2)	3 (±1)
Channels	0 (±1)	1 (±2)	3 (±1)

Note: numbers are presented as mean (±SD). ICA, independent component analysis.

**Figure 2:**
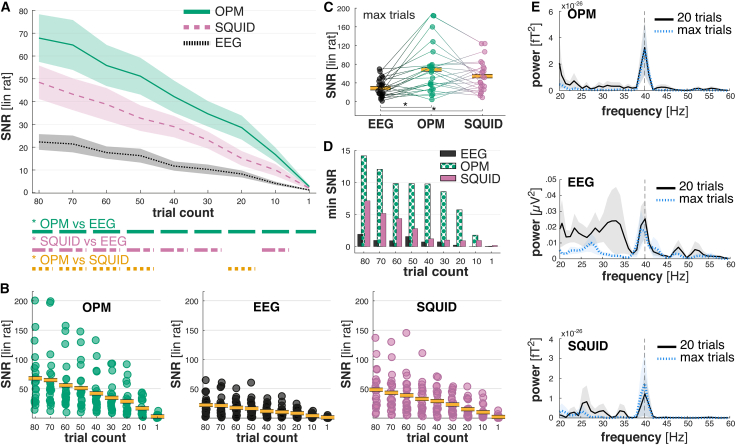
OPMs have significantly higher SNRs compared to EEG and SQUID (A) Signal-to-noise ratios (SNRs) of OPMs over trial numbers. The SNRs of OPMs were significantly higher than those of EEG and SQUID. Even when comparing only the first trial of each individual, OPMs have a significantly higher SNR compared to EEG (*n* = 21). Post hoc analyses depicting significant contrasts between modalities are illustrated below (A) (e.g., a green solid line below 80 denotes that the contrast between OPM and EEG was significant in the 80 trial condition). Shaded areas represent mean ± SEM. (B) Illustration of individual SNR values for chronological trial numbers compared between OPM, EEG, and SQUID (*n* = 21). Yellow bold lines depict the mean. (C) When incorporating all available trials, OPMs had on average the highest SNRs, significantly higher than EEG (*n* = 23). Yellow bold lines depict the mean. (D) It is notable that the minimal SNR between measurement conditions is consistently highest for OPM, higher by a factor of ∼5–6 for EEG and ∼2 for SQUID (*n* = 21). (E) The power spectrum of the trial average compared between 20 trials and the maximum number of trials for each measurement condition. Noise in surrounding frequencies is reduced for OPMs compared to EEG and SQUID, especially with a trial limitation of 20 (*n* = 21). Shaded areas represent mean ± SEM. Note: SNR is given as linear ratio (lin rat). ∗*p* < 0.05 (statistical test: ANOVA).

The magnitude of SNR increases with trial numbers also differed between measurement modalities (interaction trial number × measurement modality: *F*_(3.25,64.93)_ = 8.71; *p* < 0.01; η^2^_*p*_ = 0.30; Figure 2A). Pairwise post hoc tests showed that the SNRs of OPM signals significantly exceeded those of EEG signals under all trial count conditions. After 20 trials, OPMs reached an average SNR that was higher than the average SNR of EEG after 80 trials and of SQUID after 40 trials. Overall, there was an increase in SNR for OPMs of up to 205% compared to EEG and of up to 40% compared to SQUID. The SNR of OPM signals was significantly higher than that of SQUID signals for 20, 50, 60, 70, and 80 trials. In addition, the SNR of SQUID signals was characterized by significantly higher SNR compared to EEG signals (for all apart from 1 and 20 trials). In general, maximum SNRs for OPM signals were 38% higher than for SQUID signals and 210% higher than for EEG signals (Figure 2B).

In a separate analysis, we allowed trial numbers to differ between measurement modalities, by choosing the maximum trial number after preprocessing. Here, SNRs of both OPM and SQUID signals were significantly higher than SNRs of EEG signals (*F*_(2,44)_ = 11.49; *p* < 0.001; η^2^_*p*_ = 0.34; Figure 2C). It is noteworthy that on average (over trial numbers 10–80), the minimum SNR was 333% higher for the OPM signal compared to the SQUID signal and 2,078% higher than the minimal SNR for EEG signals (Figure 2D). The difference between measurement modalities was sufficiently salient to be visible by manual inspection of the power spectra, especially when comparing low trial numbers to maximum trial numbers (Figure 2E).

### The phase coherence of OPMs exceeds EEG and SQUID

In addition, we compared inter-trial phase coherence (ITPC), which reflects the uniformity of phase angles^42^ across neural responses between EEG, OPM, and SQUID. To this end, we calculated a ratio of ITPC (ITPC-ratio) at 40 Hz relative to adjacent frequency bands during the phase of ASSR. While ITPC is affected by individual differences, within individuals, it is characterized by high test-retest reliability.^43^^,^^44^^,^^45^

**Figure 3:**
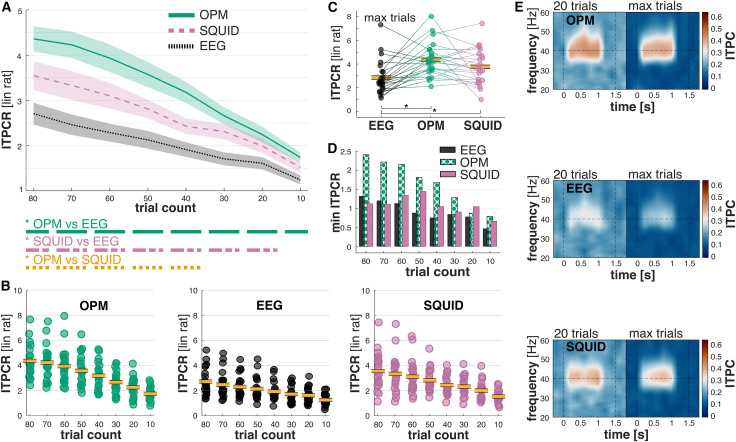
OPMs have significantly higher inter-trial phase coherence ratios compared to EEG and SQUID (A) Coherence ratios (ITPC-ratios) of OPMs over trial numbers. The ITPC-ratios of OPMs were significantly higher than those of EEG and SQUID. This difference between OPMs and EEG was already significant after 10 trials (*n* = 21). Post hoc analyses depicting significant contrasts between modalities are illustrated below (A) (e.g., a green solid line below 80 denotes that the contrast between OPM and EEG was significant in the 80 trial condition). Shaded areas represent mean ± SEM. (B) Illustration of individual ITPC-ratio values for chronological trial numbers compared between OPM, EEG, and SQUID (*n* = 21). Yellow bold lines depict the mean. (C) When incorporating all available trials, OPMs had on average the highest CRs, significantly higher than EEG (*n* = 23). Yellow bold lines depict the mean. (D) It is notable that the minimal ITPC-ratio is highest for OPM, and increased by up to ∼100% compared to EEG (*n* = 21). (E) ITPC compared between 20 trials and the maximum number of trials for each measurement condition. OPMs show the strongest ITPC-ratio at 40 Hz, even when limiting trial numbers to 20 (*n* = 21). Note: SNR is given as linear ratio (lin rat). ∗*p* < 0.05 (statistical test: ANOVA).

The ITPC-ratio differed significantly between measurement modalities (Figures 3A and 3B; *F*_(2,40)_ = 16.33; *p* < 0.001; η^2^_*p*_ = 0.45) and increased with trial numbers (*F*_(1.75,35.01)_ = 88.67; *p* < 0.001; η^2^_*p*_ = 0.82). This increase, however, differed between measurement modalities (*F*_(4.01,80.28)_ = 5.87; *p* < 0.001; η^2^_*p*_ = 0.23). Post hoc comparisons revealed that the ITPC-ratio of OPM signals was significantly higher than for EEG signals across all trial numbers. Overall, the increase in ITPC-ratio for OPMs was up to 61% compared to EEG and 23% compared to SQUID systems. Individual ITPC-ratio data points revealed that maximum ITPC-raios were comparable between OPM and SQUID (up to 7% higher for OPMs) and up to 52% higher for OPM signals compared to EEG signals (Figure 3B). Incorporating the maximum trial number for each measurement modality led to significantly higher ITPC-ratios for OPM and SQUID signals compared to EEG signals (see Figure 3C; *F*_(2,43.95)_ = 9.77; *p* < 0.001; η^2^_*p*_ = 0.31). The minimum ITPC-ratio for OPM signals was higher than for EEG and SQUID signals, with on average 76% increase in ITPC-ratio compared to EEG and 51% increase in ITPC-ratio compared to SQUID (Figure 3D). These differences are apparent even under manual inspection when comparing the ITPC-ratio between 20 trials and the maximum trial number across measurement modalities (Figure 3E). Taken together, our results indicate a stronger phase coherence in measuring neural responses across trials for OPM systems compared to EEG and SQUID systems.

### 10–16 OPM sensors versus 64 EEG electrodes versus 124 SQUID gradiometers

**Figure 4:**
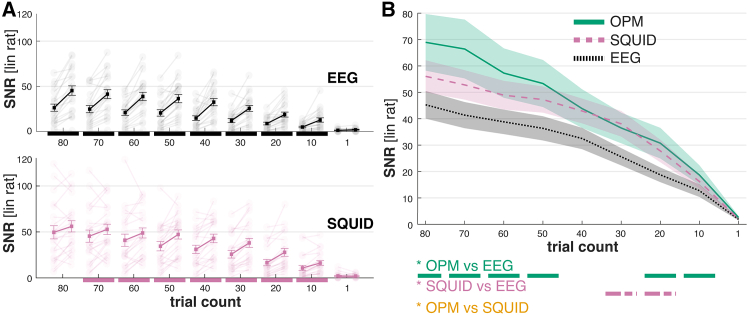
SNR increase from limited sensors to whole-head setup (A) For both EEG and SQUID, there is a significant increase in SNR from 10 sensors/electrodes to a whole-head system (56 EEG electrodes and 125 SQUID gradiometers). Apart from single trials (and 80 trials for SQUID), this difference is significant across trial counts. Post hoc contrasts with a significance level of *p* < 0.05 are marked by bold lines beneath the axes (*n* = 21). Error bars illustrate the mean ± SEM. (B) The SNRs measured with 10–16 OPMs significantly outperform those measured with 56 EEG electrodes for all trial counts but 1, 30, and 40 (*n* = 21). Compared to 125 SQUID gradiometers, the SNRs of 10–16 OPMs are comparable or better; however, this difference is not significant. Post hoc analyses depicting significant contrasts between modalities are illustrated below (B) (e.g., a green solid line below 80 denotes that the contrast between OPM and EEG was significant in the 80 trial condition). Shaded areas represent mean ± SEM. Note: SNR is given as linear ratio (lin rat). ∗*p* < 0.05 (statistical test: ANOVA).

Next, we aimed to investigate how SNR values recorded with 10–16 OPMs compare to whole-head EEG (56 electrodes) and SQUID (124 gradiometers) measurements. We observed a significant increase in SNR with the whole-head solution for both EEG (*F*_(1,20)_ = 54.30; *p* < 0.001; η^2^_*p*_ = 0.73) and SQUID (see Figure 4A; *F*_(1,20)_ = 19.79; *p* < 0.001; η^2^_*p*_ = 0.50). Increases in SNR were different across trial counts (EEG: *F*_(1.82,36.30)_ = 39.79; *p* < 0.001; η^2^_*p*_ = 0.67; SQUID: *F*_(1.99,39.79)_ = 47.22; *p* < 0.001; η^2^_*p*_ = 0.70), with significant increases present in all conditions, apart from single trials for EEG and single and 10 trials for SQUID. Additionally, SNRs increased faster with accumulating trial numbers for whole-head systems compared to limited sensor/electrode setups (interaction condition × trial count; EEG: *F*_(4.01,80.24)_ = 14.11; *p* < 0.001; η^2^_*p*_ = 0.41; SQUID: *F*_(2.21,44.24)_ = 4.57; *p* = 0.013; η^2^_*p*_ = 0.19).

We then compared SNR values between 10–16 OPM sensors and whole-head EEG and SQUID systems. Again, measurement modalities differed significantly in their SNR (*F*_(1.79,35.78)_ = 4.01; *p* = 0.031; η^2^_*p*_ = 0.17), and SNRs generally increased with trial count (Figure 4B; *F*_(1.63,32.54)_ = 47.94; *p* < 0.001; η^2^_*p*_ = 0.71). The slope of the SNR increase with higher trial counts differed between measurement modalities, as shown in a significant interaction between SNRs for measurement modalities and trial count (*F*_(4.32,86.43)_ = 2.79; *p* < 0.028; η^2^_*p*_ = 0.12). Post hoc comparisons revealed that SNRs of OPM signals were significantly higher than those of EEG signals for all trial counts apart from single trials and 30–40 trials.

## Discussion

To investigate whether OPMs can improve electrophysiological measurements, we compared 40 Hz ASSRs obtained with EEG and conventional MEG (SQUID) and OPMs in healthy participants. So far, there has been no comprehensive comparison between OPM, EEG, and SQUID. Although previous studies indicated superior performance of OPMs compared to EEG and comparable performance to SQUID, the findings were based on relatively small sample sizes and provided inconsistent evidence.^2^^,^^3^^,^^22^^,^^29^^,^^30^^,^^31^^,^^32^

Our findings demonstrate that OPMs, a new generation of quantum sensors, surpass EEG and conventional SQUID in SNR and ITPC-ratio during 40 Hz ASSR. SNRs of OPM signals were up to 205% higher compared to EEG signals and up to 40% higher compared to SQUID signals. Additionally, ITPC-ratio was higher for OPMs, with an increase of up to 61% compared to EEG and up to 23% compared to SQUID. Our findings emphasize the potential of OPMs to advance non-invasive human neuroimaging, as previous studies demonstrated the importance of increased SNR for accurate estimation of functional connectivity, source reconstructions, and biomarkers.^46^^,^^47^^,^^48^

Additionally, accumulating data suggest a need for higher SNR to differentiate neuronal generators,^14^^,^^15^ which, due to mixing of sources, produce similar electric potentials in EEG recordings.^49^^,^^50^ Additionally, there are simulations which propose that OPMs may enhance differentiation between layer-specific cortical activity.^51^

Here we show that OPMs provide significantly higher ITPC-ratio than EEG and SQUID, reflecting increased consistency in the acquisition of the evoked signal over trials. Decreased ITPC-ratio for EEG compared to OPMs is likely caused by limited SNR or mixing of sources.^14^^,^^15^ Because ITPC-ratio partly depends on amplitude, particularly for the SQUID recording acquired in a separate session, an extent of observed differences could also stem from higher signal amplitudes for OPMs compared to SQUID measurements.^3^

A prior study investigated evoked potentials, but unlike in our results, the authors found equivalent or lower SNRs for OPM compared to SQUID signals.^27^ These discrepancies may be explained by sensor-to-signal distance, which affects SNR, especially when sensor density is unequal. To prevent this bias, we matched sensor numbers and positions between systems and applied a spatial filter^38^ to maximize the representation of the signal of interest across sensors. Consistent with the previous study, we found no SNR differences across modalities when using only the highest SNR sensor/electrode, which generally led to strongly reduced SNRs, indicating that single sensors do not exhaust the full potential of each measurement method (Figure S1).

Importantly, OPM signals reached average SNRs with significantly fewer trials (20 vs. 80) than EEG and even SQUID (20 vs. 40). Even on the single-trial level, the difference in SNR was significant. This has implications for task designs in both basic neuroscience clinical populations. Longer measurements in pediatric as well as clinical populations can be difficult, in part because of movements.^5^^,^^52^ Additionally, in severe cases of epilepsy, the localization accuracy of the epileptic focus, which directly depends on SNR, can affect the success of surgery and the chance of seizure freedom.^53^^,^^54^^,^^55^ In both cases, reduced measurement time as well as increased SNRs achieved by OPMs may provide a significant improvement to the current standard procedure.

This is further underlined by the fact that minimal SNRs for OPMs were several fold higher than those for EEG and SQUIDs, showing that in recordings where noise was relatively high, OPMs were better at capturing the signal of interest. Furthermore, increased SNR for single trials improves the application of brain-computer interfaces, which have gained importance in clinical application, such as for brain-state-dependent neuromodulation.^56^ These findings are corroborated by recent studies, which indicated improved performance for an OPM-based brain-computer interface compared to SQUID.^18^^,^^57^ Additionally, we showed that 10–16 OPM sensors were sufficient to surpass SNRs acquired with a whole-head EEG and achieve on average higher SNRs acquired with a whole-head SQUID system. Additional studies validating OPMs in other settings, incorporating additional frequency ranges and sensory modalities, are required. Movement through transient background fields can create slow-frequency noise, which may introduce an additional source of noise for OPMs. Regarding the measurement of slow-frequency activity, promising evidence has been provided for an auditory-mismatch paradigm, where 5 OPM sensors provided similar SNRs to EEG and SQUID systems,^58^ indicating a possible generalizability of our results to other settings and paradigms. These outcomes encourage the development of flexible measurement systems as a potential bedside application with mobile shielding, outperforming the current standard of EEG in clinical settings.

Taken together, our findings demonstrate increased SNR and ITPC-ratio for OPMs compared to the current gold-standard methods EEG and SQUID during 40 Hz ASSR, even when sensor and trial numbers are limited. While more research is needed to validate the application of OPMs in different settings, such as other sensory modalities, frequency ranges, and deeper sources, our results indicate that OPMs have the potential to significantly advance non-invasive neuroimaging, which could be relevant for neuroscience, brain-computer interfaces, and precision diagnostics and therapy.

### Limitations of the study

It should be noted that there are certain caveats in comparing different measurement modalities. Neuronal responses to auditory stimulation, in general, may be more easily recorded with OPM and SQUID systems compared to EEG, based on the source orientation in Heschl’s Gyrus.^40^^,^^45^^,^^59^ Furthermore, the SQUID system in this study comprised gradiometers, which improve SNRs by reduction of non-neuronal noise.^60^ The OPM sensors applied in this study, on the other hand, comprised two measurement directions each, which increases channel density, benefitting the application of spatial filters, such as canonical correlation.^38^^,^^39^ This effect may be most relevant for our subset analysis, which limited sensor/electrode count to 10 per modality. The 10 sub-sensors were selected based on topographic matching, introducing uncertainties that may affect sensor performance metrics. Additionally, OPM sensors are closer to the signal source than SQUID sensors, resulting in higher amplitudes of the recorded signal but potentially also increased noise from unrelated brain sources.^24^ In general, it is difficult to assess the effects of orientation differences OPM and SQUID sensors may have. Moreover, the EEG-OPM recording was performed upright, while the SQUID recording was performed in supine position. There are differences in neuronal signal strength as well as spontaneous neural activity between these positions.^61^^,^^62^^,^^63^ For visual paradigms, it could be shown that the cortical cerebrospinal fluid layer decreases in thickness in a supine position, which brings sensors closer to the signal source and increases signal strength.^62^ It is currently unclear how auditory steady-state responses differ between body postures, and we cannot rule out that differences in positions affect SNRs. Furthermore, participants show more movement in the upright position, which introduces additional sources of noise. For this reason, we refrained from analyses of the baseline period in each trial, as we specifically allowed participants to move and blink between trials, resulting in pronounced noise in EEG and OPM (upright) compared to SQUID (supine) recordings. Lastly, it is noteworthy that the SQUID recording took place in a room with reduced shielding compared to the optimal environment for our EEG-OPM recording, which affects the remaining background field and potential environmental noise sources such as from car or train traffic. Especially for OPM recordings, where body movements cause OPM sensors to transition through background fields, a lower shielding factor could introduce increased low-frequency noise. However, due to the fact that SQUID systems are static and therefore not affected by gradients in the background fields, the applied shielding is sufficient to suppress environmental noise in our setting.^41^

## Resource availability

### Lead contact

Requests for further information and resources should be directed to and will be fulfilled by the lead contact, Marion Brickwedde (marion.brickwedde@ptb.de).

### Materials availability

This study did not generate new unique materials or reagents.

### Data and code availability

• All data reported in this paper will be shared by the lead contact upon request.
• This paper does not report original code.
• Any additional information required to reanalyze the data reported in this paper is available from the lead contact upon request.

## Acknowledgments

We thank the 10.13039/501100001659DFG for funding this project and ANT-Neuro for cooperating with us in the modification of their EEG-cap. Additionally, we would like to thank our colleague Dr. Tineke Grent-t'-Jong, who provided a lot of theoretical and practical support in the project. Lastly, we would like to express our gratitude to Anna Benedict, Carlotta Preller, Katharina Stammkötter, and Fabian Symanowski, who supported data acquisition. This project is funded by the 10.13039/501100001659Deutsche Forschungsgemeinschaft (DFG; project no. 460785001).

## Author contributions

Conceptualization, M.B., T.S., and P.J.U.; resources, T.S., P.K., and P.J.U.; data curation, M.B. and P.A.; software, M.B. and P.A.; formal analysis, M.B.; supervision, T.S., P.K., and P.J.U.; funding acquisition, T.S. and P.J.U.; validation, P.A., T.S., P.K., and P.J.U.; investigation, M.B. and P.A.; visualization, M.B.; methodology, M.B.; writing – original draft, M.B.; project administration, M.B., T.S., and P.J.U.; writing – review and editing, P.A., T.S., P.K., and P.J.U. All co-authors have read and approved the final version of the manuscript.

## Declaration of interests

The authors declare no competing interests.

## STAR★Methods

### Key resources table

**Table:** 

REAGENT or RESOURCE	SOURCE	IDENTIFIER
**Software and algorithms**		

MATLAB vR2020b	Mathworks	https://www.mathworks.com/products/matlab.html
FieldTrip v20221223	Oostenveld et al.^64^	https://www.fieldtriptoolbox.org/download/
PTB DAQ System	Elzenheimer et al.^65^	N/A
Presentation v 21	NeuroBehavioral Systems	https://neurobs.com/menu_presentation/menu_features/features_overview
PsychoPy	Open Science Tools	https://www.psychopy.org/download.html
eego	ANT Neuro	https://www.ant-neuro.com/

**Other**		

Human Participants	This study	N/A

### Experimental model and study participant details

We recruited N = 23 healthy volunteers between 18 and 55 years (mean = 35 years ± 10; 11 = female; migrant background = 5; see Table 1 for details; race and ethnicity information was not collected). As this study focused on intra-individual comparisons between brain imaging technologies, it is not expected that demographic attributes such as race, gender or ethnicity will have impacted the results of this study.

Participants were recruited via university mailing lists and online advertising. At the end of the experiment, participants received monetary compensation (10 € /h). The study protocol was approved by the Ethics Committee of the Charité University Hospital, Berlin (EA2/004/20), and in accordance with the Declaration of Helsinki. All participants provided written informed consent.

### Method details

#### Data acquisition

EEG data was acquired with an ANTNeuro cap (Bandwidth limited by sampling frequency: 500 Hz/2; Noise amplitude spectral density (NASD): 0.4–2.5 μV/ √ Hz), OPM data was acquired with a QuSpin QZFM system 2^nd^ generation (Bandwidth: < 130 Hz; NASD: 15 – 30 ft/ √ Hz) and SQUID data was acquired with a Yokogawa axial gradiometer system (Bandwidth limited by sampling frequency: 4000 Hz / 2; NASD: 2–3 ft/√ Hz). For pictures of the setup see Figure S2.

For parallel EEG/OPM recordings, participants were seated inside an 8+1-layer magnetically shielded room at Physikalisch-Technische Bundesanstalt (PTB), Berlin.^65^^,^^66^ The recordings were performed using a modified 56-electrode cap (equidistant layout) by AntNeuro with tailor-made OPM holders (see Figure 1A). Additional AgCl ring electrodes were placed next to the eye (electrooculogram) and at the left and right earlobe (reference and ground). Electrode positions were cleaned with Nuprep skin preparation gel and OneStep abrasive gel, keeping impedances below 10 kΩ. For the combined recording of EEG and OPM, we estimated the magnetic field interference which may arise from EEG electrode and cables. With a B-field < 5 ∗ 10^-18^T, the expected interference should be too small to affect OPM-measurements. The interference was estimated as follows: B = $\frac{\mu_{0}\;*\;I}{2\;*\;\pi \;*r}$, where *μ*_0_ = 1.3 ∗ 10^-6^*N*/*A*^2^ (constant vacuum permeability), r > 5 mm (conservative estimation of the distance of the centre of the gas cell to cables/electrodes), I = $\frac{U}{R}$, U < 100 μV (conservative estimation of measured EEG-signals), R > 1 GOhm (impedance of the ANT eego amplifier).

The number of available OPM sensors differed between participants, ranging from 10 to 16 dual-channel OPM sensors, which were positioned over left and right temporal regions (see Table 2; see Figure 2). Online OPM recordings were sampled at 4000 Hz and recorded with the PTB data acquisition system DAQ-system.^65^ EEG recordings were sampled at 500 Hz and were recorded with the ANT eego software. Visual stimuli were presented via a shielded projector.^67^

SQUID data was acquired in a separate session using a Yokogawa system with 124 gradiometers. This session of the experiment took part in a 2+1-layer shielded room. Participants were lying in supine position, while visual targets or distractors were projected onto a screen. Online SQUID recordings were sampled at 4000 Hz. To avoid confounds due to repetition and learning effects, half of the participants performed the EEG/OPM session first, while the other half started with the SQUID session.

For all measurements, auditory stimulation was applied utilizing an Etymotic® sound system and transmitted into the shielded room via tubes, which were connected to in-ear-plugs (Doc’s Promolds).

#### Paradigm

Prior to recordings, individual hearing thresholds were assessed utilizing a staircase procedure. During the main experiment, participants focused on a central fixation cross while sounds were played in the background which consisted of 1000 Hz carrier tones, either amplitude modulated at 40 Hz (100 trials), at 20 Hz (4 trials) or at 60 Hz (4 trials), presented in random order (see Figure 1C). Trials modulated at 20 Hz and 60 Hz were accompanied by arrows either pointing to the left or to the right. Participants were asked to press a button when arrows pointing to the right appeared, and to ignore arrows pointing to the left. Stimulation was performed via PsychoPy^68^ for the EEG/OPM recordings and with Presentation® software (Version 21.0, Neurobehavioral Systems, Inc., Berkeley, CA, www.neurobs.com) for the SQUID recordings.

#### Data preprocessing

The preprocessing steps were identical across measurement modalities. Data were segmented into 4s epochs starting 1s prior to ASSR stimulation and down sampled to 500 Hz. EEG data were re-referenced to an average reference. To remove line-noise, data was notch-filtered between 49 and 51 Hz (original dataset). All filters applied to the data were implemented in the Fieldtrip Toolbox (zero-phase Butterworth IIR filter, 4^th^ order, forward-reverse filtering). For visual artifact inspection, pseudo-datasets were created and filtered between a) 110 and 140 Hz to manually identify trials with strong muscle artifacts and between b) 0.5 and 80 Hz to identify other movement artifacts, as well as noisy or flat channels. Additionally, a semi-automatic algorithm was used to visually identify trials with strong deviations in amplitude variance. All identified bad trials and channels were removed from the original dataset.

As the amount of removed trials directly impacts signal-to-noise ratios, it could be argued that removed trials should be identical between EEG and OPM. However, as the measurement modality may directly impact the number of trials that need to be removed and could therefore be considered an important factor in the evaluation of the method, we decided to preprocess EEG and OPM data separately (see Table 3 for number of removed trials/channels/components per modality).

For the application of an independent component analysis (ICA), a pseudo-dataset was filtered between 5 and 40 Hz. Manually identified eye-blink-, eye-movement-, and heartbeat-components were removed from the original dataset, which was then filtered between 5 and 60 Hz.

To retrieve channel weights which optimally represent the measured signal, a spatial filter was created using canonical correlation.^38^^,^^39^ To this end, channel weights which maximized the covariance between the bandpass filtered single trials with the bandpass filtered average signal during the time of stimulation (0.25 s – 1 s; bandpass filter: 39-41 Hz) were calculated for each individual and modality separately. The data matrix was then multiplied with the channel weights and summarized over channels to create a virtual channel best representing the 40 Hz neuronal response. As canonical correlation is insensitive to polarity, an algorithm was devised which identified local minima and maxima of the average over all participants and channels (without canonical correlation). These datapoints were then applied to individual virtual channel datasets. If the average over all local minima was higher than the average over all local maxima, the data was multiplied by -1. Afterwards, the average signal was inspected manually per participant to confirm the polarity based on event-related fields/potentials.

In some analyses, to achieve optimal comparability, we limited sensor/electrode numbers to 10 for all measurement modalities as a last step before applying canonical correlation. For SQUID and OPM, auditory 40 Hz activity can best be measured over temporal areas. Accordingly, we chose 5 lateral positions above the left and right ear, which best approximated matched positions for a standard head size. Due to volume conduction, EEG signals from temporal dipoles cannot be measured well over temporal areas but show maximal activation over frontocentral areas. Accordingly, we chose 10 electrode positions covering these areas, which showed on average the highest amplitude response to auditory 40 Hz stimulation (see Figure 1C for layouts of all modalities and the chosen electrodes/sensors).

All preprocessing steps and analyses were performed in MATLAB R2020b and the fieldtrip toolbox, version 20221223.^64^

### Quantification and statistical analysis

To assess SNR between measurement modalities, spectral power of the trial average and inter-trial phase coherence (ITPC) were computed. Spectral power was calculated between 0.25 and 1 s using a sliding Hanning taper with a dynamic duration of 7 cycles (frequency range: 1 to 60 Hz, step size: 0.5 Hz). ITPC was calculated using a sliding Gaussian wavelet with a dynamic duration of 7 cycles. The frequency ranged from 1 to 60 Hz in a step size of 0.1 Hz and the temporal resolution was set to 0.25 between -1 s and 2 s.

SNRs were calculated by comparing 40 Hz spectral power and ITPC with adjacent frequencies. As such, SNRs of the power data constituted a comparison between 40 Hz and the mean across a noise frequency-range between 20 and 38 Hz as well as 42–48 Hz, which was defined as: SNR = $\frac{power\;\left(40\;Hz\right)}{average\;power\;\left(noise\;frequency\;range\right)}$. For ITPC data, the noise frequency range was limited to 20-30 Hz to avoid frequency-smearing of signal into the noise range.

To compare SNR estimates for different trial numbers, we successively reduced the number of trials inserted into the power/ITPC estimation used for SNR-calculation. Trials were always chosen in chronological order to best represent real experiments with, for instance, only 10 trials. For this analysis, we removed 2 participants as both had less than 70 trials left after preprocessing in at least one measurement condition.

Percent increase between SNRs of measurement modalities was calculated as *SNR*_*inc*_= $\frac{\left(V_{\mathrm{mod}\;1}-V_{\mathrm{mod}\;2}\right)}{V_{\mathrm{mod}\;1}}*100$, where V_mod1_ denotes the SNR value of modality 1 and V_mod2_ denotes the SNR value of modality 2.

Statistical differences between modalities were assessed using repeated-measures ANOVA utilizing Tukey’s Honest Significant Difference as post-hoc assessment. To assess post-hoc contrasts over different trial numbers, separate two-factorial repeated-measures ANOVAS between pairwise measurement modalities were calculated. Sphericity of the factor variables was investigated with Mauchly’s test. Cases which violated the assumption of sphericity were corrected using the Greenhouse Geisser method, and adjusted p-values and degrees of freedom are reported. As an effect size, η^2^_p_ was calculated as

$$\eta_{P}^{2}=\frac{Sum\;of\;Squares\;\left(effect\right)}{Sum\;of\;Squares\left(effect\right)+Sum\;of\;Squares\;\left(residuals\right)}\text{.}$$
