# Supplementary material for: Optically pumped magnetometers enhance neuroimaging performance—An EEG, OPM, and SQUID-MEG study
Source: iScience. 2026 Mar 26;29(5):115489. doi: 10.1016/j.isci.2026.115489 (PMC13091735; doi:10.1016/j.isci.2026.115489)
Supplement: Document S1. Figures S1 and S2 [file mmc1.pdf]

iScience, Volume *29*

Supplemental information

Optically pumped magnetometers enhance neuroimaging performance—An EEG, OPM, and SQUID-MEG study

Marion Brickwedde, Paul Anders, Peter Krüger, Tilmann Sander, and Peter J. Uhlhaas

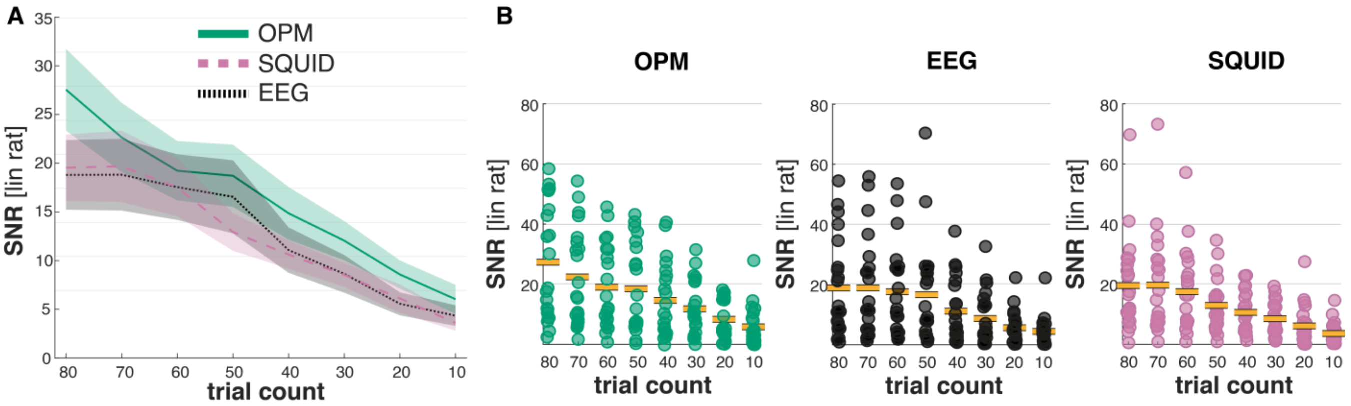

Figure S1. SNR comparison between OPM, EEG and SQUID-systems for single highest SNR sensor/electrode – related to discussion. (A) On average, OPM-signals show the highest SNR values across trial counts, however, differences between measurement modalities are not significant (n = 21). Shaded areas represent SEMs. (B) A closer look at individual SNR-values illustrates that SNR-values across modalities are comparable when selecting a single sensor with the highest SNR for each individual. SNR-values on average are decreased by a factor of ~2-3 compared to the application of spatial filters (see Fig. 2). *Note: SNR is given as linear ratio (lin rat).* * < p.05

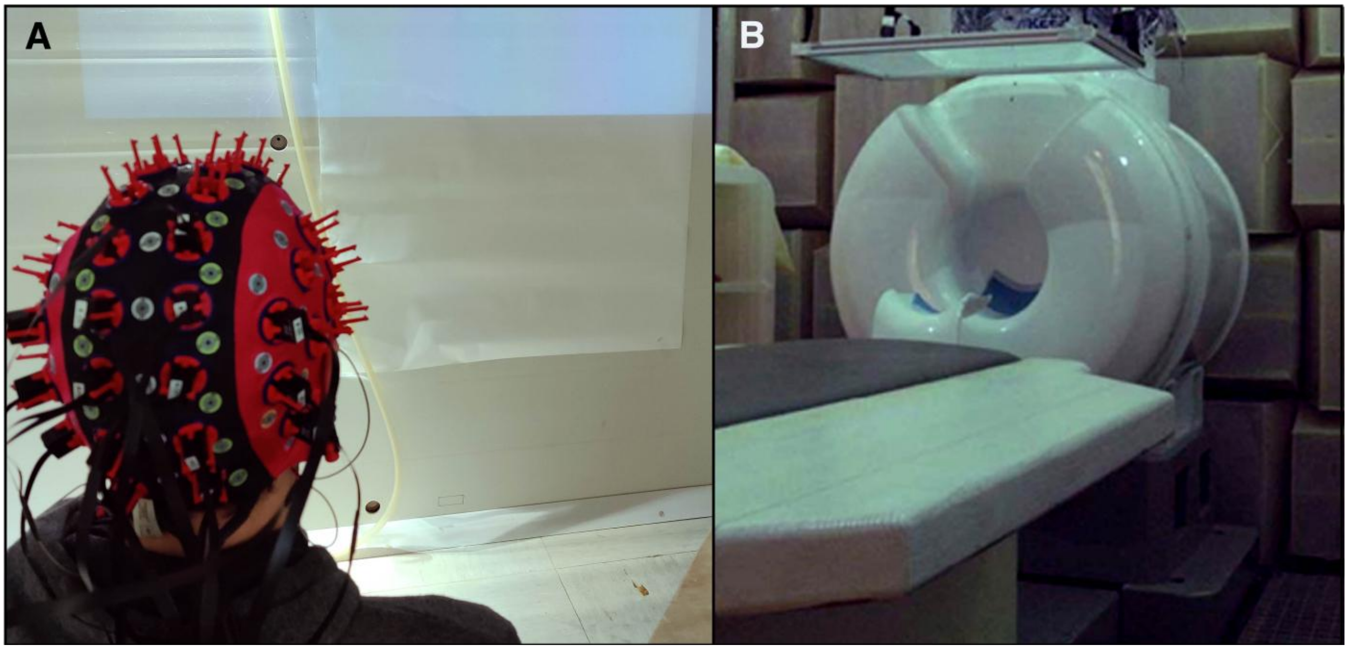

Figure S2. Exemplary fotos of the EEG, OPM and SQUID setup – related to STAR methods. (A) The participant is seated upright in an 8+1 layer shielded room wearing an EEG-cap from ANTNeuro with OPM sensors positioned over occipital areas (in the present study, OPM sensors were positioned over temporal areas). (B) The Yokogawa SQUID system used in this study. Participants lay down on a bed and are moved into the dewar.
